# Developmental, Reproduction, and Feeding Preferences of the *Sitobion avenae* Mediated by Soil Silicon Application

**DOI:** 10.3390/plants12050989

**Published:** 2023-02-21

**Authors:** Xiaoru Wang, Weiwei Li, Jia Yan, Yi Wang, Xingyan Zhang, Xiaoling Tan, Julian Chen

**Affiliations:** 1 State Key Laboratory for Biology of Plant Diseases and Insect Pests, Institute of Plant Protection, Chinese Academy of Agricultural Sciences, Beijing 100193, China; 2 Key Laboratory of Economical and Applied Entomology of Liaoning Province, College of Plant Protection, Shenyang Agricultural University, Shenyang 110866, China

**Keywords:** silicon, wheat, *Sitobion avenae*, life table parameters, feeding preference

## Abstract

Silicon occupies an important position in the nutrient requirements of wheat. It has been reported that silicon enhances plant resistance to phytophagous insects. However, only limited research has been carried out on the effects of silicon application to wheat and *Sitobion avenae* populations. In this study, three silicon fertilizer concentrations were treated for potted wheat seedlings, including 0 g/L, 1 g/L, and 2 g/L of water-soluble silicon fertilizer solution. The effect of silicon application on the developmental period, longevity, reproduction, wing pattern differentiation, and other vital life table parameters of the *S. avenae* were determined. The cage method and the Petri dish isolated leaf method were used to determine the effect of silicon application on the feeding preference of the winged and wingless aphid. The results showed silicon application had no significant effect on the aphid instar of 1–4; although, 2 g/L silicon fertilizer prolonged the nymph stage and 1 and 2 g/L of silicon application all shortened the adult stage and reduced the longevity and fertility of the aphid. Two instances of silicon application reduced the net reproductive rate (*R*_0_), intrinsic rate of increase (*r_m_*), and finite rate of increase (λ) of the aphid. A 2 g/L silicon application prolonged the population doubling time (*t_d_*), significantly reduced the mean generation time (*T*), and increased the proportion of winged aphids. The results also demonstrated that the selection ratio of winged aphids in wheat leaves treated with 1 g/L and 2 g/L silicon was reduced by 8.61% and 17.88%, respectively. The number of aphids on leaves treated with 2 g/L silicon was significantly reduced at 48 and 72 h of aphids released, and the application of silicon to wheat was detrimental to the feeding preference of *S. avenae*. Therefore, the application of silicon at 2 g/L to wheat has an inhibitory effect on the life parameters and feeding preference of *S. avenae*.

## 1. Introduction

Wheat (*Triticum aestivum* L.) is one of the cereal crops with the largest sown area, the widest distribution, and the highest production in the world. It is a staple food for populations in many countries and regions, as well as the third largest grain crop in China; therefore, the level of wheat production is crucial to human food security and sustenance [1,2]. Wheat aphids are generally regarded as the most serious pests in cereals in most regions of the world, with a very high reproductive capacity, a short life cycle, and a polymorphic nature, and are therefore involved in a high frequency of outbreaks [3]. *Sitobion avenae* is one of the main pests in the wheat-producing areas of China. It not only directly sucks the sap of wheat, such as stems and leaves, causing physical damage to the wheat by losing nutrients but also transmits plant viruses, thus causing damage such as failure to produce ears and appear unsaturated grains. This pest has become a major threat to wheat cultivation, causing yield reductions of 15–30%, which have even reached 40–60% in severe years [4,5]. Due to the serious damage caused by wheat aphids in wheat fields, the control of these pest mainly depends on chemical insecticides; however, the overuse of insecticides leads to the development of resistance and, at the same time, results in environmental pollution and the contamination of grains, posing a serious threat to the ecological environment and human safety and health [6,7]. Therefore, it is urgent to develop new eco- and environmentally friendly pest control measures.

Silicon is the second most abundant element in the Earth’s crust, making up 26.4% of the Earth’s mass [8]. Jones [9] reported that silicon was found in plants. Silicon is a beneficial element for plants and plays an important role in regulating the interrelationships between plants and other organisms, resisting adversity, and alleviating stress [10,11]. Wheat is a cereal crop with high silicon content and, throughout its life, has a relatively high demand for silicon. Studies have reported that silicon can enhance plant resistance to phytophagous insects by thickening the plant’s physical defense barrier and inducing the expression of the plant’s resistance genes. The application of silicon fertilizer is one of the alternative strategies for pest control [12,13,14]. Silicon deposited in plant tissues increases its hardness and abrasion resistance and reduces the selectivity of phytophagous insects and plant digestibility [15,16,17,18]. There are many studies on the application of silicon fertilizer in rice but few studies concerning its application on wheat. The feeding ability, reproductive capacity, and longevity of *Chilo suppressalia* and *Nilaparvata lugens* on silicon-treated rice were reduced compared to non-silicon-treated rice [19,20,21]. It was found that brown planthopper adults and rice stem borer preferred feeding significantly less on rice treated with high silicon than on plants with low silicon content, which may be related to callus deposition in the plant [22,23]. Leaf spraying and the soil application of silicon reduced the preference of aphids for maize and enhanced aphid resistance in maize [24]. High silicon content in wheat and sorghum reduced the fecundity and longevity of the wheat aphids and reduced their damage to plants [25,26,27]. Silicon application enhances crop resistance to *Schizaphis graminum*, and resistant crop varieties had significantly higher levels of silicon than controls [28]. Silicon application significantly reduced the selectivity, survival, and fecundity of *S. graminum* on certain crops [29,30]. Silicon fertilization is able to reduce colonization by alates, enhance non-preference resistance, and reduce population growth of apterous *S. avenae* in wheat plants [12]. Therefore, additional silicon fertilization offers the potential to enhance wheat resistance to wheat aphids. Although some people have focused on the effect of silicon on wheat resistance [12,13,26,27], there are few reports concerning the exact effect of applying different concentrations of silicon fertilizer to wheat and the development, reproduction, and feeding preferences of *S. avenae*. We would also like to know whether the selection rate is different after different release times. In addition, the effect of silicon fertilizer application concentration on different pests on different crops is also of great concern. The research of the effect of silicon fertilizer on the body weight and wing type differentiation of aphids is limited.

In this study, we precisely control the concentration of silicon fertilizer. By setting different concentrations of silicon fertilizer, the effects of silicon application on the fitness (including developmental duration, longevity, reproduction, wing pattern differentiation, and other vital life table parameters) and the preference of the *S. avenae* were conducted, which likely provide a theoretical basis for reducing pesticide usage and guiding rational fertilizer application. Moreover, the results may enrich the control strategy of wheat aphids, providing both a safe, economical, and effective method for the integrated control of wheat aphids and a basis for the development of new green control technologies for wheat aphids.

## 2. Materials and Methods

### 2.1. Wheat 

The tested wheat variety was AiKang 58. The wheat seeds were soaked in distilled water at 4 °C for 24 h and then germinated at 25 °C for 48 h. The seeds were planted in plastic pots (10.5 cm high, 10.5 cm top diameter, and 8 cm bottom diameter) filled with a 2:1 ratio of vermiculite to turf (Sinopharm Chemical Reagent Co., Ltd., Shanghai, China). Five seeds were sown in each pot. After 5 days, the seeds were germinated, and one seedling was kept in each pot. A total of 100 mL of water was poured into each pot every 2 days thereafter. 

### 2.2. Insect

*S. avenae* were collected with a trap net at the Xinxiang Experimental Station, Institute of Plant Protection, Chinese Academy of Agricultural Sciences, Xinxiang County, Henan Province (113°78′ E, 35°15′ N). This strain was reared for more than five generations in a rearing room at 20 ± 1 °C, with a relative humidity of 75–80%, and a photoperiod ratio of L:D = 16 h:8 h. Nymphs within 24 h of the first production were selected for the following experiments.

### 2.3. Silicon Treatments

After germination, the silicon was applied by spraying 50 mL of silicon solution on the leaves of wheats in each pot. The above operation was repeated every 5 days. Three silicon treatments, including control (no silicon application 0 g/L), 1 g/L, and 2 g/L of silicon (alkaline water–soluble silicon fertilizers with ≥55% SiO_2_).

For each treatment, 0.29 g of nitrogen, 0.48 g of phosphate, and 0.36 g of potash (46.4% N for urea, 44% P_2_O_5_, 16% N for diammonium phosphate, and 60% K_2_O for potash) per kg of soil were applied in a single application of urea, diammonium phosphate, and potash chloride as a base fertilizer, which was mixed well after application. The pots were placed in an environmental greenhouse at a humidity of 70–80%, a temperature of 20 ± 1 °C, and a photoperiod which was the same as that described in Section 2.2. After 35 days of seedling growth, wheat plants from different treatment pots were selected for the trial.

### 2.4. Effects of Silicon Application on the Nymph Developmental Period and Survival Rate of S. avenae

The effect of silicon application on the development and reproduction of the aphid was investigated by comparing aphid development period and weight on wheat with and without silicon treatment. When wheat had grown for 35 days, a wingless adult *S. avenae* were placed on the second leaves of each wheat shaft and covered with a plastic ecological box (2.7 cm × 2.7 cm × 2.7 cm) in order to prevent aphids from escaping. After the adult aphids produced nymphs, only one nymph was left on the leaf, and the others were removed. The development period and survival rate of the aphids were observed and recorded every day at 9 am, 3 pm, and 8 pm. The experiment consisted of 3 replicates with 10 samples per replicate.

### 2.5. Effects of Silicon Application on the Aphids Reproduction and Longevity of S. avenae

Aphids produced within 24 h of the treatment described in Section 2.4 were placed on wheat treated with the corresponding silicon fertilizer. Wheat age, aphid type, soil treatment, and wheat aphid treatments for this test were similar with that described in Section 2.4. After the nymphs grew to adulthood, their lifespan was recorded and the number of aphids produced was observed and recorded every 24 h, until they died; during this period the new nymphs were removed. The experiment consisted of 3 replicates with 10 samples per replicate.

### 2.6. Effects of Silicon Application on the Adult Weight and Wing Pattern Differentiation

Aphids produced within 24 h of the treatment described in Section 2.4 were placed on wheat treated with the corresponding silicon fertilizer to determine body weight and wing pattern differentiation. Fifteen first instars of aphids were attached to each of the second leaves of non-silicon-treated and silicon-treated wheat and covered with a plastic ecological box until the nymphs became adults, at which point the wing pattern was recorded and the total weight of the 15 adults was recorded using an electronic balance (EX225DZHAD (accuracy 0.001 mg), OHAUS Instruments Changzhou Ltd., Changzhou China), within 24 h. The proportions of winged aphids and their adult weight were counted every 15 aphids and replicated times.

### 2.7. Effects of Silicon Application on the Feeding Preference of the Wingless S. avenae on Hosts 

For the wingless aphid non-selectivity test, a 20 cm diameter, 25 cm high Petri dish with small holes in the sides and white filter paper in the bottom was used. A centrifuge tube (3 cm long, 0.5 cm diameter) with wheat leaves inserted inside was placed on one side of a large Petri dish. The centrifuge tube contains distilled water mixed with 0.2 μL of a 10^−6^ mol/L solution of 6-benzylaminopurine solution (which inhibits leaf chlorophyll breakdown and preserves greenness and prevents aging), and it was plugged with cotton. 

Wheat leaves were selected from the first fully expanded leaves of different treatments at 35 days of age, cut off, and placed in the centrifuge tube. A total of 45 wingless adult aphids were released at the other side of the Petri dish, which was on the opposite side of the leaf. The Petri dish was sealed with perforated parafilm and placed flat on the experimental bench. The experiment was carried out in an artificial climate chamber at a temperature of (22 ± 2 °C), with the relative humidity and light conditions as described in Section 2.2. The number of aphids on each cut leaf was counted at 24, 48 and 72 h after the release of aphids. Each treatment was replicated five times.

### 2.8. Effects of Silicon Application on the Feeding Preference of the Winged S. avenae on Hosts 

The in vitro wheat shrouding cage method was used to determine the effect of silicon application on the host selectivity of the winged *S. avenae*. One 30-day-old wheat plant was selected from each treatment and three plants with different silicon treatments were placed in a conical bottle containing nutrient solution, arranged equidistantly in an equilateral triangle with a distance of 80 cm between the two bottles, and covered with a rectangular 120 mesh nylon cage (length × width × height: 100 cm × 90 cm × 50 cm). In the center of the triangle, a Petri dish (9 cm in diameter and 2 cm high) was placed with 30 winged aphids of the same age. The number of winged aphids on each treatment of wheat was recorded 48 h after the release of the aphids, and the winged aphid host hobby was assessed. One cage was one replicate, and a total of 15 replicates were set up under natural light environmental conditions, with the temperature and relative humidity as described in Section 2.2.

### 2.9. Data Analysis 

The life table parameters, including net reproductive rate (*R*_0_), mean generation time (*T*), intrinsic rate of increase (*r_m_*), and finite rate of increase (λ) of the aphid, were calculated according to the life table parametric calculation method [31,32], where X is the aphid age stage expressed in days, *L_x_* is the survival rate of stage X, and *M_x_* is the average aphid production of adult aphids at age stage X. The parameters of the life tables of *S. avenae* were calculated using the jackknife technique. To obtain stable and precise estimates, we used 100,000 bootstraps.

Net reproductive rate *R*_0_ = ∑*l_x_m_x_*;

Mean generation time *T* = ∑*xl_x_m_x_*/*R*_0_;

Intrinsic rate of increase *r_m_* = ln*R*_0_/*T*;

Finite rate of increase λ = $e^{r_{m}}$;

Population doubling time *t_d_* = ln2/*r_m_*.

Percentage data from the experiment were subjected to a square root inverse metaphor transformation and then tested. All data were subjected to a one-way analysis of variance (ANOVA) for the significance of differences between treatments using Tukey’s HSD tests (*p* = 0.01) in R statistical software (R development Core Team, 2009). All graphs were produced using GraphPad Prism biostatistics software (GraphPad Software Inc., San Diego, CA, USA).

## 3. Results

### 3.1. The Effect of Silicon Application on the Developmental Duration of the S. avenae

Feeding on wheat treated with 1 g/L and 2 g/L silicon had no significant effect on the first instar (*F* = 0.746, *df* = 2, 42, *p* = 0.481), second instar (*F* = 0.259, *df* = 2, 42, *p* = 0.773), third instar (*F* = 2.145, *df* = 2, 42, *p* = 0.13), and fourth instar (*F* = 2.516, *df* = 2, 42, *p* = 0.093) of *S. avenae*, but the nymph duration gradually increased with increasing silicon application (*F* = 11.732, *df* = 2, 42, *p* = 0.00009 < 0.01) and differed significantly between two silicon treatments. The nymph period was 2.1% and 4.8% longer than the control with the 1 g/L and 2 g/L silicon treatments, respectively. Silicon application significantly shortened the life span of the adult (*F* = 12.609, *df* = 2, 42, *p* = 0.00005 < 0.01), which was 7.7% and 11.3% shorter, respectively, than the control with the 1 g/L and 2 g/L silicon treatments (Table 1).

### 3.2. The Effect of Silicon Application on the Growth and Development of the S. avenae

**Figure 1:**
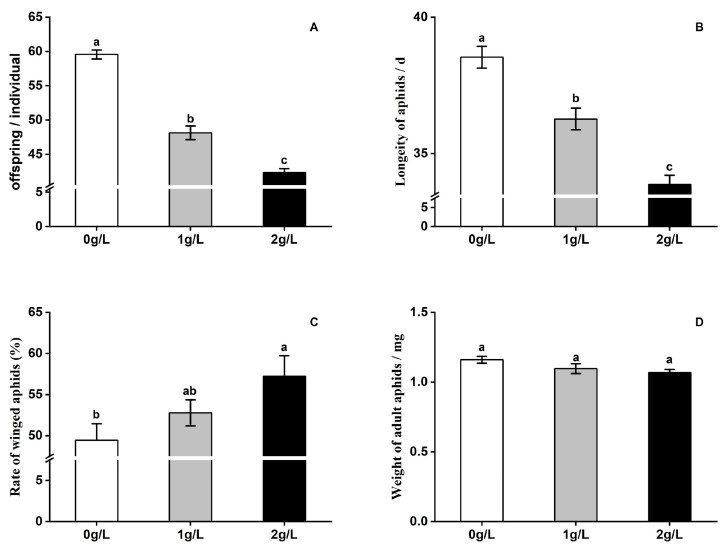
Effect of silicon addition to wheat plants (AK58) on *S. avenae* development, survival, and reproduction. (**A**): fecundity, (**B**): longevity of aphids, (**C**): rate of winged aphids, (**D**): weight of adult aphids. Different letters over the bars indicate a significant difference (*p* < 0.01).

Feeding on wheat with 1 g/L and 2 g/L silicon treatments significantly reduced the offspring No. (*F* = 133.964, *df* = 2, 42, *p* < 0.01) (Figure 1A) and longevity (*F* = 37.953, *df* = 2, 42, *p* < 0.01) (Figure 1B) of *S. avenae*. The 1 g/L silicon treatment had no significant effect on the rate of the winged aphid, but the 2 g/L silicon treatment significantly increased the rate of winged aphid on wheat compared to the control (*F* = 3.575, *df* = 2, 15, *p* = 0.054) (Figure 1C). There was no significant effect of silicon application on the weight of adult aphids (*F* = 2.869, *df* = 2, 15, *p* = 0.088) (Figure 1D).

### 3.3. The Effect of Silicon Application on Thelife Table Parameters of the S. avenae

Compared to the non-silicon control, the finite rate of increase (λ) (*F* = 13.470, *df* = 2, 42, *p* < 0.01), net reproductive rate (*R*_0_) (*F* = 132.094, *df* = 2, 42, *p* < 0.01), and intrinsic rate of increase (*r_m_*) (*F* = 13.393, *df* = 2, 42, *p* < 0.01) of *S. avenae* were significantly lower than those of the non-silicon control. The doubling time (*T*_2_) of the *S. avenae* population was significantly longer (*F* = 12.917, *df* = 2, 42, *p* < 0.01). There was no significant effect on the mean generation time (*T*) of *S. avenae* during the 1 g/L silicon treatment compared to the control, but it was significantly reduced during the 2 g/L silicon treatment (*F* = 9.954, *df* = 2, 42, *p* < 0.01) (Table 2).

### 3.4. Effect of Silicon Application on No-Selectiity of the Wingless S. avenae

**Figure 2:**
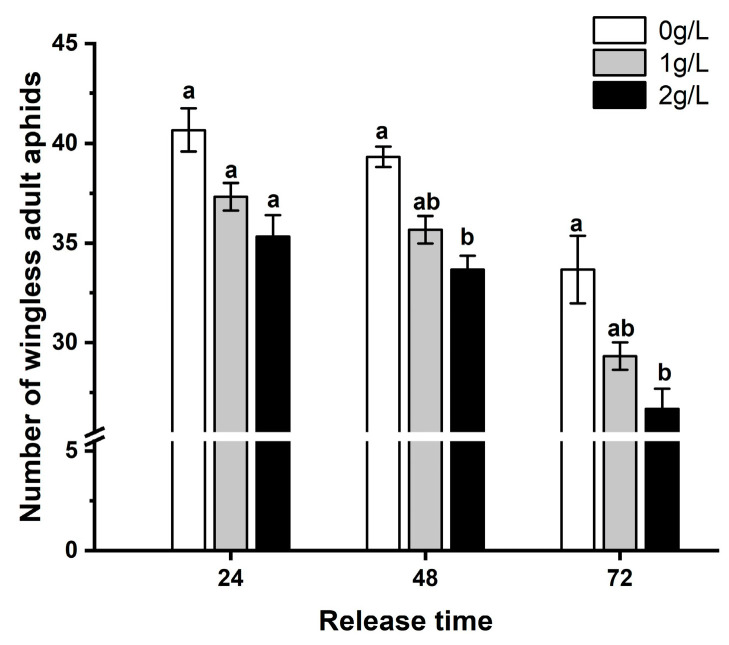
The number of *S. avenae* on isolated wheat leaves treated with different silicon concentrations. Bars with different letters are significantly different (Tukey’s HSD test, *p* = 0.01).

There was no significant effect of 1 g/L silicon concentration-treated wheat leaves on the number of aphids compared to non-silicon treated leaves within 24 h (*F* = 3.403, *df* = 2, 15, *p* = 0.06), 48 h (*F* = 4.827, *df* = 2, 15, *p* = 0.024), and 72 h (*F* = 8.915, *df* = 2, 15, *p* = 0.0028 < 0.01) of aphid release. There was no significant effect of 2 g/L silicon on aphid numbers on wheat leaves at 24 h of aphid release compared to non-silicon treated leaves, but a significant reduction of 9.93% and 14.41% in aphid numbers on silicon-treated leaves at 48 and 72 h, respectively. Mean aphid numbers on isolated leaves at 24 h, 48 h, and 72 h of aphid release were significantly reduced by 9.97% and 15.84% for the 1 g/L and 2 g/L silicon treatments, respectively, compared to leaves that did not receive silicon (*F* = 11.495, *df* = 8, 45, *p* < 0.01) (Figure 2).

### 3.5. The Effect of Silicon Application on the Selectivity of the Winged S. avenae

**Figure 3:**
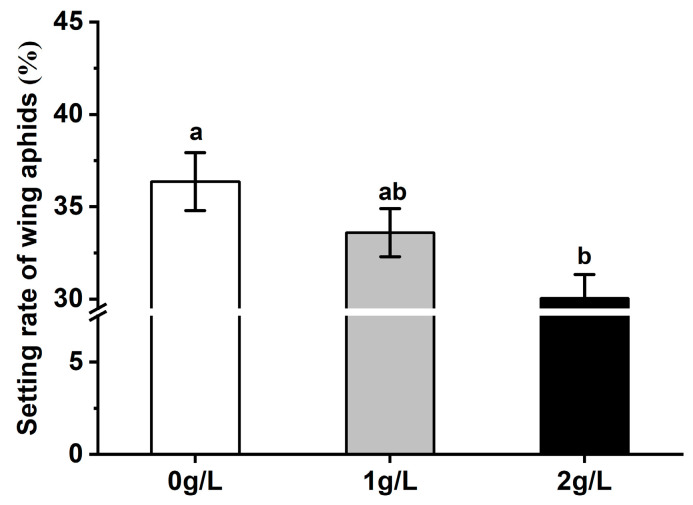
Effects of different silicon treatments on wheat plants’ host acceptance of winged *S. avenae*. Bars with different letters are significantly different (Tukey’s HSD test, *p* = 0.01).

Silicon treatment significantly affected the selectivity of the winged *S. avenae* regarding wheat leaves. The settling rate of *S. avenae* on the leaves of wheat treated with 1 g/L silicon was 8.61% lower than that of the control, and no significant difference as compared with the control was seen. However, the settling rate of winged aphids on leaves treated with 2 g/L silicon was significantly lower than that of the control (*F* = 5.187, *df* = 2, 42, *p* = 0.0097 < 0.01) (Figure 3).

## 4. Discussion

The application of silicon has a direct or indirect effect on plant resistance, including enhancing plant resistance, reducing the growth and reproduction of phytophagous insects, feeding selectivity, etc. [33,34]. Wheat is one of the silicon-loving crops and has a high demand for silicon during its growth and development. Wheat tissues and cells actively take up silicon and deposit it in order to enhance their hardness, creating some resistance to insect attack and reducing crop damage and losses [28]. However, different concentrations of silicon fertilizer have different effects on wheat and wheat aphids. Therefore, in our study, we focused on the effects of different concentrations of silicon on the development, reproduction, and preference of wheat aphids.

In this study, the nymph development stage of *S. avenae* was significantly prolonged and the adult longevity was significantly shortened after feeding on wheat treated with a 2 g/L concentration of silicon, which was able to potentially enhance the strength and hardness of plant tissues, increase the physical resistance of wheat to the aphids, and hinder the feeding behavior of the aphid, thus affecting its growth, development, and reproduction. This is similar to the results of the study on significantly shortened the adult longevity of *S. avenae* on silicon treated wheat [13], and the apparent prolongation of the larval stages of *Cnaphalocrocis medinalis* and *N. lugens* on silicon-treated rice [21,35]. However, this study found that the prolongation of the nymph stage of *S. avenae* after 2 g/L silicon application to wheat was inconsistent with studies on the developmental period of *Schizaphis graminum*, possibly related to inconsistencies in the different concentrations and methods of applying silicon fertilizer [30]. In this study, silicon application reduced the longevity and aphid production of *S. avenae* and enhanced the wing pattern differentiation rates of *S. avenae*. In the study by Dias [13] on the effect of silicon application on *S. avenae*, the application mode and concentration of silicon were different from those in our study, but their results were consistent with those obtained by us [13], which is also similar to previous studies that showed that silicon application reduced the viability and reproduction rate of the *S. graminum* and *Sogatella furcifera* [17,25]; however, the effect of silicon application on the body weight of adult aphids was not significant. This is inconsistent with previous studies that silicon application is able to reduce the body weight of insects and may be related to the different insect species [21].

Life table parameters are an important basis for assessing the impact of plants on insect population dynamics. They are able to comprehensively reflect and describe the development, reproduction, survival, and longevity of insects [36]. This study found that silicon application with 1 g/L and 2 g/L significantly reduced the *R*_0_, λ, and *r_m_* of *S. avenae* and that 2 g/L prolonged the *t_d_*. This is similar to the results of studies on the effects of silicon application on the life table parameters of *N. lugens*, *Myzus persicae*, and *C. medinalis* [21,35,37]. Therefore, certain concentrations of silicon application enhance the wheat resistance of wheat plants to *S. avenae* and suppress the development and reproduction of *S. avenae*.

Insect non-preference is one of the characteristics of plant resistance to herbivorous insects. Non-preference refers to the ability of plants to reduce pest damage by producing defense signaling molecules or their own physiological and biochemical properties that prevent insects from favoring plants for feeding, reproduction, and settlement. It has been shown that silicon application to wheat and rice is able to reduce the settling of *S. graminum, C. medinalis*, and *N. lugens* pests [20,21,25,35]. In this study, both the non-selective tests of wingless aphid and the selective tests of winged aphids demonstrated that a high concentration of silicon fertilizer treatment deterred aphids from selecting wheat, which has similar results to the preferences of *S. graminum* regardomg silicon and non-silicon treatments [26]. Similar results have been obtained in other crop research systems [13,17,38], which may also be due to silicon-fertilized wheat leaves. A mechanical barrier is formed on the cell walls of the tissues, making it difficult for the pests to penetrate and pierce the outdoor oral needles, affecting their feeding choices and behavior [39]. In future studies, the EPG technique could be used to investigate differences in feeding behavior and details of the *S. avenae* on wheat treated with different silicon concentrations. Previous studies have found that the inhibition of phytophagous pests by silicon may be related to secondary plant metabolites. When plants are subjected to adversity stress, silicon can initiate the activity of protective enzymes, hydrogen peroxide, soluble proteins, and other related defense substances in the body, forming direct or indirect defense safeguards to hinder pest feeding and reproduction [40,41,42,43]. At the same time, we found that in the non-selective experiment with aphids without wings, different concentrations of the silicon treatment had no significant effect on aphid selectivity at the early stage of aphid release. The high concentration did not affect aphid colonization until 48 h after application. This resistance generally comes from non-volatile resistance substances in the plant, which may affect the feeding and digestion of herbivorous insects [5].

Studies have shown that the silicon enhancement of plant resistance to phytophagous insects may be related to the channeling of signaling substances, such as salicylic acid and jasmonic acid in plants. Silicon induces the production of protective enzymes, soluble proteins, and other insect-resistant substances in plants, resulting in a defense response that inhibits the growth and development of phytophagous insects [12,44,45]. The mechanisms underlying the inhibition of population growth and unfavorable feeding selection and preference of the *S. avenae* by silicon application are currently poorly understood in this experiment. For example, it is not clear how insect attacks induce the production of various defense enzymes and defense-related hormones, such as protective enzymes (SOD, POD), salicylic acid, and jasmonic acid; whether silicon is involved in the up-regulation of the role of defense-related genes in jasmonic acid and salicylic acid biosynthesis or whether silicon is a combination of other biotic stress factors that together induce defense responses and enhance the plant. Whether silicon is involved in the up-regulation of defense-related genes in jasmonic acid and salicylic acid biosynthesis or whether other biotic stress factors combine to induce defense responses and enhance plant resistance to insects needs further investigation and clarification.

In summary, the results of this paper show that the application of 2 g/L silicon fertilizer to wheat prolongs the nymph period; reduces aphid longevity and aphid production; decreases the intrinsic rate of increase, finite rate of increase, and net reproductive rate; and shortens the mean generation time and extends population doubling time and that 2 g/L silicon application to wheat has a reducing effect on the number of wingless aphids settling on leaves and a repelling effect regarding the feeding choice of winged aphid after release for 48 h. It is believed that silicon fertilizer has good application prospects in integrated pest management.

## Figures and Tables

**Table 1:** Effects of silicon treatments on the developmental period of *S. avenae*.

Instar	The Developmental Duration		
	0 g/L Control	1 g/L Si Treatment	2 g/L Si Treatment
**1st instar (d)**	2.593 ± 0.008 a	2.621 ± 0.007 a	2.644 ± 0.007 a
**2nd instar (d)**	1.918 ± 0.009 a	1.925 ± 0.009 a	1.949 ± 0.007 a
**3rd instar (d)**	2.003 ± 0.008 a	2.061 ± 0.012 a	2.137 ± 0.015 a
**4th instar (d)**	2.338 ± 0.017 a	2.429 ± 0.015 a	2.542 ± 0.018 a
**Nymphs (d)**	8.852 ± 0.017 b	9.037 ± 0.014 b	9.273 ± 0.017 a
**Adults (d)**	29.582 ± 0.149 a	27.296 ± 0.166 b	25.094 ± 0.173 c

Data are expressed as mean ± SE. Data in a line followed by different letters are significantly different (Tukey’s HSD test, *p* = 0.01).

**Table 2:** Life table parameters of *S. avenae* on different silicon-treated wheats.

Population Parameters	Silicon Treatments		
	0 g/L (Control)	1 g/L	2 g/L
Intrinsic rate of increase (*r_m_*)	0.378 ± 0.0005 a	0.364 ± 0.0006 b	0.361 ± 0.0008 b
Finite rate of increase (*λ*)	1.231 ± 0.0006 a	1.223 ± 0.001 b	1.219 ± 0.0008 b
Net reproductive rate (*R*_0_)	59.566 ± 0.1694 a	48.233 ± 0.2594 b	42.267 ± 0.1447 c
Mean generation time (*T*)	10.807 ± 0.0119 a	10.621 ± 0.2200 a	10.390 ± 0.3419 b
Population doubling time (*t_d_*)	1.833 ± 0.0084 b	1.901 ± 0.0131 b	1.925 ± 0.1077 a

Data are expressed as mean ± SE. Data in a line followed by different letters are significantly different (Tukey’s HSD test, *p* = 0.01).

## Data Availability

Not applicable.
